# Detecting the left atrial appendage in CT localizers using deep learning

**DOI:** 10.1038/s41598-025-99701-6

**Published:** 2025-05-02

**Authors:** Aydin Demircioğlu, Denise Bos, Anton S. Quinsten, Lale Umutlu, Oliver Bruder, Michael Forsting, Kai Nassenstein

**Affiliations:** 1 Institute of Diagnostic and Interventional Radiology and Neuroradiology, University Hospital Essen, Hufelandstraße 55, 45147 Essen, Germany; 2 Department of Cardiology and Angiology, Contilia Heart and Vascular Center, Elisabeth-Krankenhaus Essen, Klara-Kopp-Weg 1, 45138 Essen, Germany; 3 Faculty of Medicine, Ruhr University Bochum, 44801 Bochum, Germany

**Keywords:** Radiation safety, CT localizer, Coronary CT angiography, Heart, Deep learning, Anatomy, Cardiology, Medical research

## Abstract

Patients with cardioembolic stroke often undergo CT of the left atrial appendage (LAA), for example, to determine whether thrombi are present in the LAA. To guide the imaging process, technologists first perform a localizer scan, which is a preliminary image used to identify the region of interest. However, the lack of well-defined landmarks makes accurate delimitation of the LAA in localizers difficult and often requires whole-heart scans, increasing radiation exposure and cancer risk. This study aims to automate LAA delimitation in CT localizers using deep learning. Four commonly used deep networks (VariFocalNet, Cascade-R-CNN, Task-aligned One-stage Object Detection Network, YOLO v11) were trained to predict the LAA boundaries on a cohort of 1253 localizers, collected retrospectively from a single center. The best-performing network in terms of delimitation accuracy was then evaluated on an internal test cohort of 368 patients, and on an external test cohort of 309 patients. The VariFocalNet performed best, achieving LAA delimitations with high accuracy (97.8% and 96.8%; Dice coefficients: 90.4% and 90.0%) and near-perfect clinical utility (99.8% and 99.3%). Compared to whole-heart scanning, the network-based delimitation reduced the radiation exposure by more than 50% (5.33 ± 6.42 mSv vs. 11.35 ± 8.17 mSv in the internal cohort, 4.39 ± 4.23 mSv vs. 10.09 ± 8.0 mSv in the external cohort). This study demonstrates that a deep learning network can accurately delimit the LAA in the localizer, leading to more accurate CT scans of the LAA, thereby significantly reducing radiation exposure to the patient compared to whole-heart scanning.

**Supplementary Information:**

The online version contains supplementary material available at 10.1038/s41598-025-99701-6.

## Introduction

Cardioembolic stroke (CES) is a common disease from which around 4–5 million suffer yearly^1^. A common cause of CES is atrial fibrillation, which is common in the elderly^2^. In these cases, the stroke results from the embolization of thrombi that form in the left atrial appendage (LAA)^3^. Systemic anticoagulation or implantation of an LAA occluder is used to prevent CES in patients with atrial fibrillation^4^. In the clinical routine, transesophageal echocardiography (TEE) is utilized to determine whether thrombi are present in the LAA or to assess the LAA anatomy prior to occluder implantation^5^. Unfortunately, TEE comes with several inconveniences: The patient must be fasting, which usually means not eating for eight hours and not drinking for two hours prior to the procedure. Swallowing the TEE device is uncomfortable, so the examination is generally performed under sedation. Although the overall risk of TEE is low, there is a risk of reactions to the drugs used for sedation (e.g., nausea and shortness of breath) and rupture of the esophagus, which is potentially life-threatening.

Computed tomography (CT) is a less invasive and more comfortable approach to assessing the LAA. Despite its good diagnostic accuracy, the relatively high radiation exposure of CT scans has prevented it from becoming established in clinical routine for the evaluation of the LAA.

**Fig. 1:**
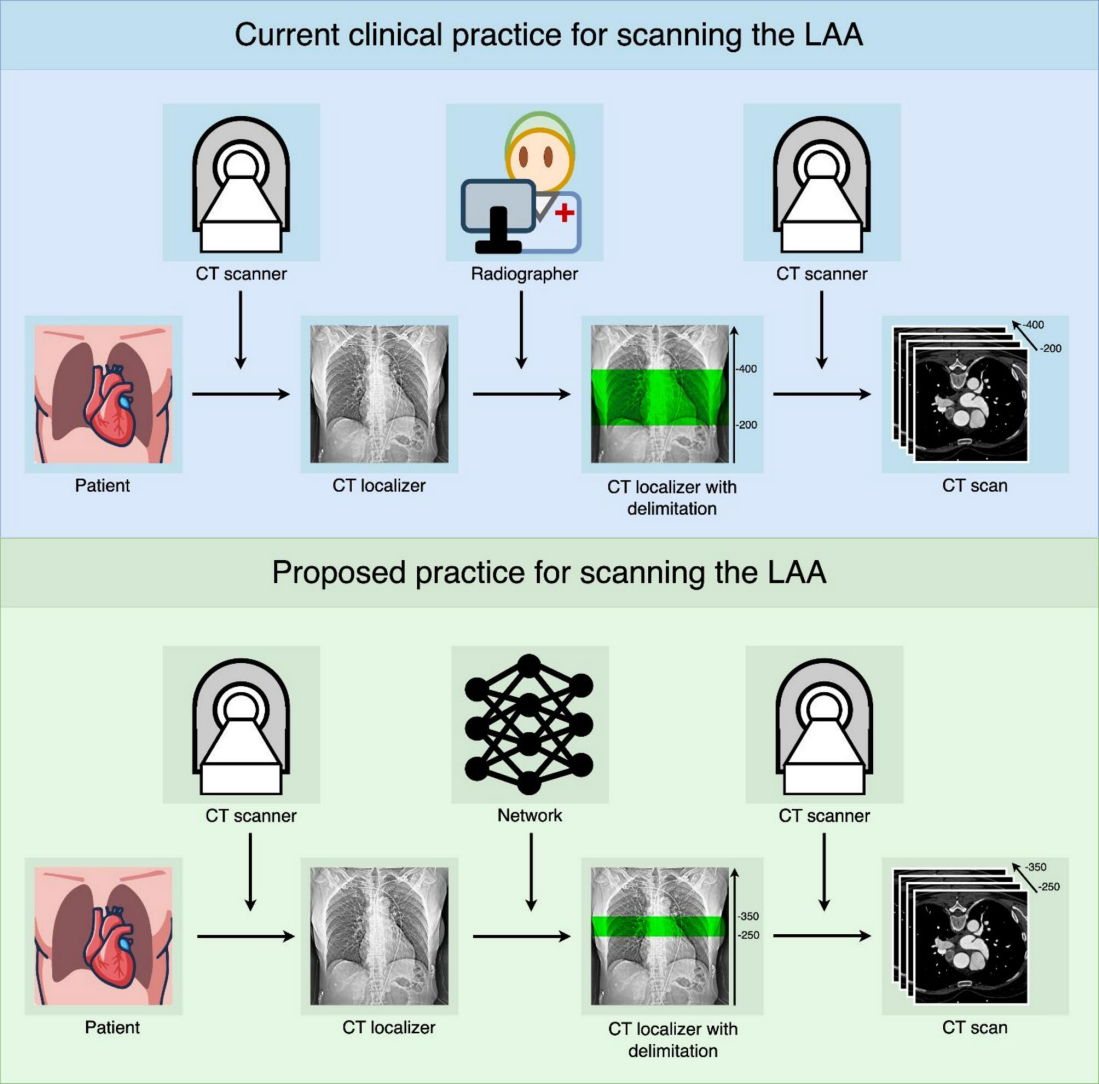
Current and proposed clinical practice for LAA CT imaging workflow. Comparison of current clinical practice (top) with the proposed deep learning-based approach (bottom) for CT imaging of the left atrial appendage (LAA). In conventional practice, manual delimitation of the localizer is performed; however, due to the anatomical variability of the LAA and the absence of clear landmarks, whole-heart delimitation is frequently necessary, resulting in increased radiation exposure. The proposed neural network enables precise LAA delimitation, thereby reducing radiation exposure while maintaining diagnostic accuracy. *ROI* region of interest.

Until now, a CT scan has been planned by manually delimiting the region of interest on a localizer, which is a low-dose overview image typically in a frontal projection. However, when the region lacks well-defined landmarks, technologists often delimit a larger area to ensure that no relevant structures are missed in the CT scan^6,7^. Indeed, due to varying anatomical shapes, there are no clear landmarks to delimit the LAA. In practice, therefore, nearly the entire heart is scanned, resulting in unnecessarily high radiation exposure (Fig. 1).

Deep learning could be a solution to minimally delimiting the LAA in the localizers, as it has been shown that, in some cases, it can perform on par with humans and, given enough data, can even perform better^8,9^. Therefore, a deep network may be able to delimit the LAA in the localizer, thereby reducing the CT scan area and, consequently, improving patient safety (Fig. 1). However, deep learning is highly dependent on data quality, and since the LAA is barely visible in the localizer, this could obstruct the application of deep networks. One solution to this problem is to use the subsequent coronary computed tomography angiography (CCTA) scan, where the LAA can be easily delimited. Transferring the annotation in the CCTA back to the corresponding localizer provides a ground truth that also accounts for patient body motion (for example, due to breathing), from which the network can learn to delimit the LAA without relying on fixed landmarks.

The present study aims to develop and evaluate the feasibility of fully automated delimitation of the left atrial appendage in the localizer, thereby reducing the overall scan area relative to whole-heart scanning using deep learning methods. The study was carried out using training and validation datasets from one institution and an independent test dataset from a nearby institution. Four well-established neural networks were trained to predict the left atrial appendage (LAA) region, with their performance evaluated using accuracy, the Dice coefficient, and mean absolute error. To assess potential clinical utility, the expected effective dose (ED) was calculated, accounting for scenarios in which network predictions might fail, necessitating whole-heart scans. Safety margins were systematically adjusted during training to account for variability in patient positioning and movement, optimizing scan area delimitation while ensuring complete LAA imaging. The model’s clinical applicability and potential to reduce patient radiation exposure were subsequently evaluated in the test sets.

## Results

### Patient data

**Fig. 2:**
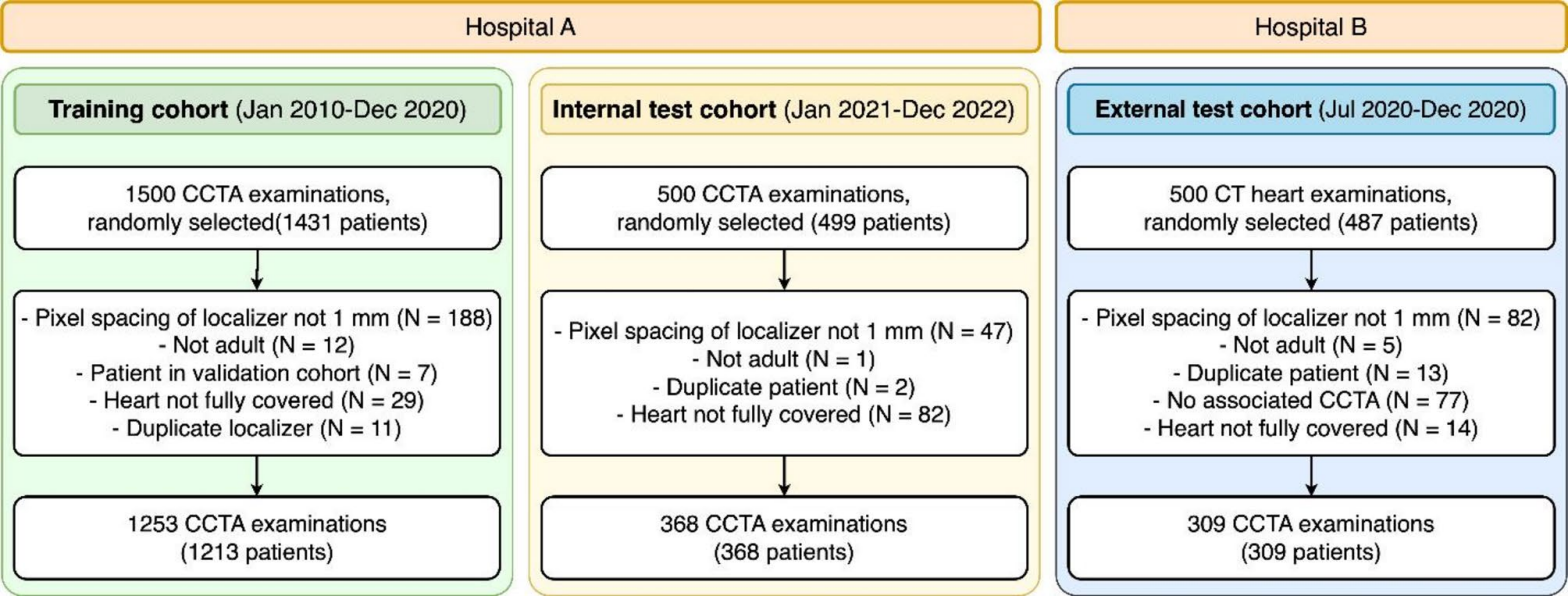
Patient flowchart with inclusion and exclusion criteria. Three independent cohorts were used. From Hospital A, we used a chronological split to obtain a training cohort and an internal test cohort. It was ensured that there was no overlap between these two cohorts to avoid any bias. In addition, the test sets contained only one scan per patient. In total, 1253 scans from 1213 patients were used for model training and 677 scans from 677 patients were used for model testing.

**Fig. 3:**
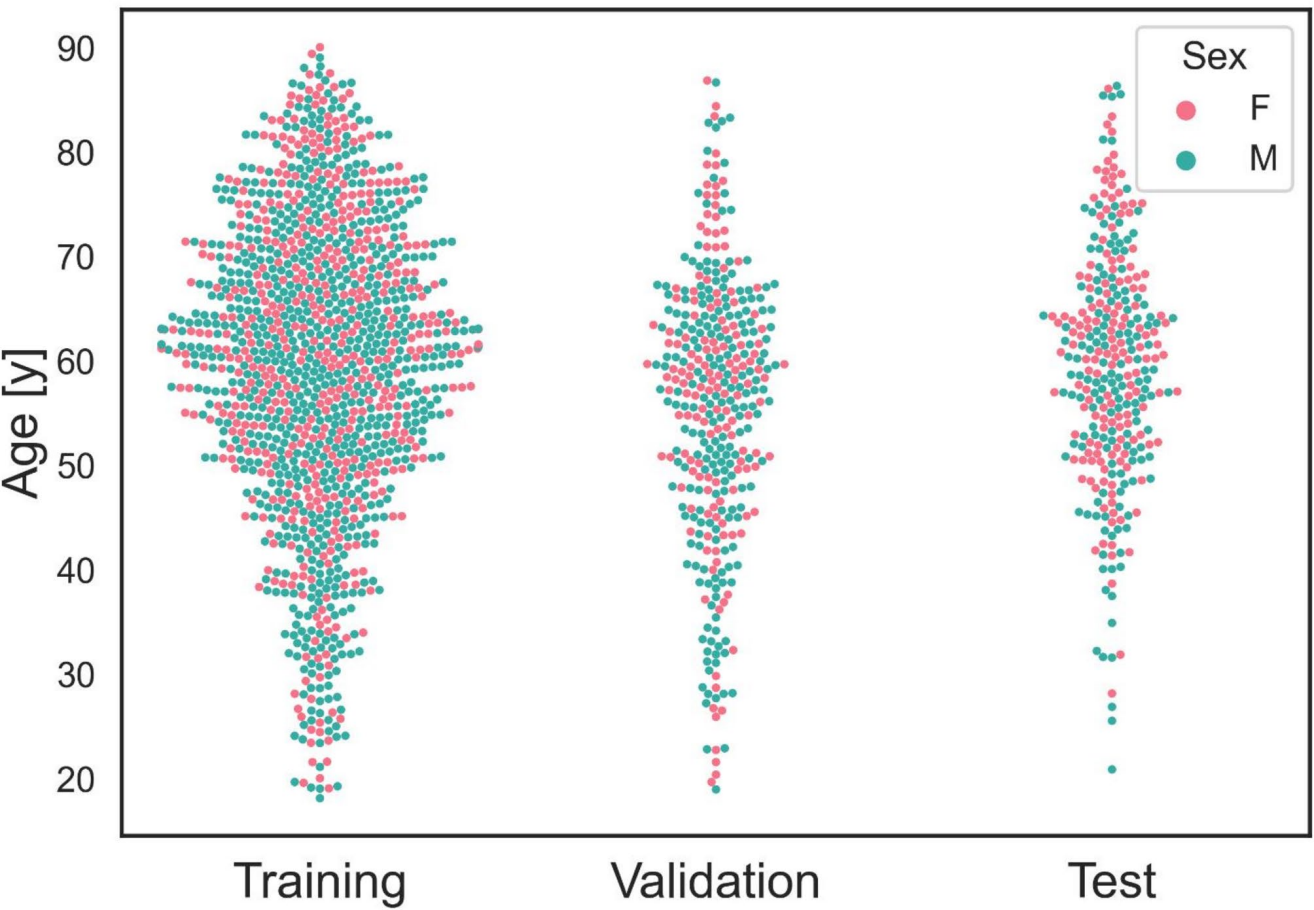
Graphical plot of patient age and sex. Distribution of patient age and sex in all three independent cohorts. Female patients are represented by a red dot, male patients by a blue dot. There is a small but significant difference in age between the training and internal cohorts, but not between the training and external cohorts. Similarly, there is no difference in gender between the training and internal cohorts, but a significant difference between the training and external cohorts.

Overall, 1253 localizers (of 1213 patients) were collected for training and validation (Fig. 2). The internal test cohort comprised 368, and the external test cohort 309 localizers, with one localizer per patient in each test cohort. The mean age of all patients was 58.6 ± 13.7 years (range 18.2–90.1 years), with 791 females and 1099 males (Table 1; Fig. 3). While no large difference is seen between the distributions of sex (*p* = 0.76) between the training and the internal test set, a difference was visible in age (*p* < 0.001). Contrarily, a difference in sex between the training and the external test set was observed (*p* = 0.002) but not in age (*p* = 0.56).

**Table 1:** Demographics of the patient cohorts.

	All (*N* = 1890)	Training (*N* = 1213)	Internal test (*N* = 368)	External test (*N* = 309)
Female	41.9%	40.0%	41.0%	50.2%
Male	58.1%	60.0%	59.0%	49.8%
Age (year)	58.6 ± 13.7	59.3 ± 14.3	55.5 ± 13.1	59.4 ± 11.4

The acquisition of the localizers was performed most often with tube voltage fixed at 120 kV and tube current at 20 or 35 mA (Table 2). In a few cases, a retrospective gated CCTA was used. The collimation size was 128 × 0.6 mm, and the rotation speed was 0.28/s.

**Table 2:** CT scanners used for the acquisition of the CT localizers.

Scanner	All (*N* = 1930)	Train (*N* = 1253)	Internal test (*N* = 368)	External test (*N* = 309)	Tube voltage	Tube current
SOMATOM Definition Force	1000	633	367	0	120 kV	20 mA
SOMATOM Definition Flash	706	396	1	309	120 kV	35 mA
SOMATOM Definition AS/AS+	199	199	0	0	120 kV	36 mA
Other	25	25	0	0	80–140 kV	20–60 mA

Scanners with less than 50 examinations were gathered into the “Other” group.

### Validation

**Fig. 4:**
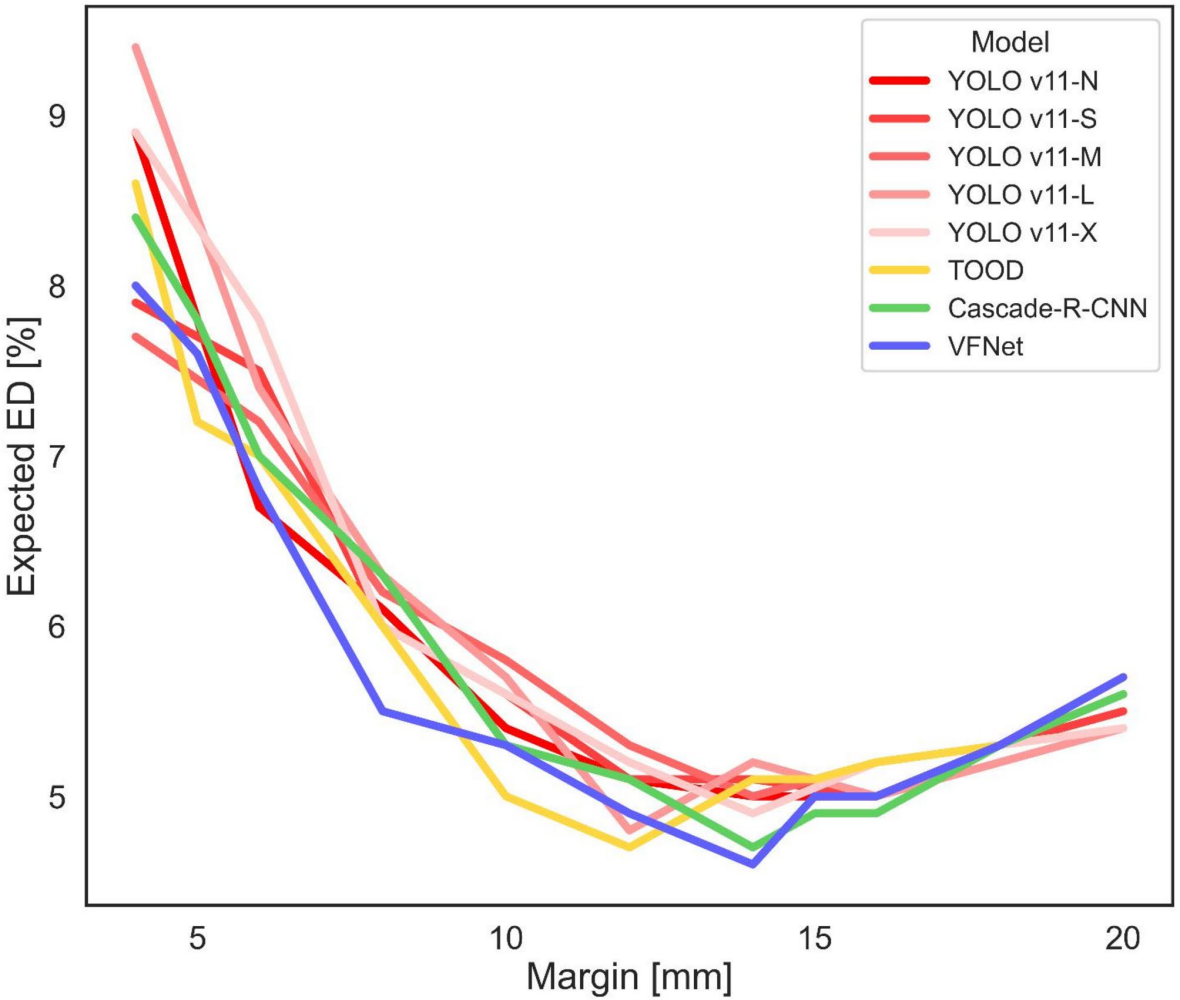
Plot of mean expected effective radiation dose against the safety margin during validation. Due to the patient’s movement (e.g., breathing), the network was trained with a safety margin added to both sides of the annotation. The size of the margin was treated as a hyperparameter. During validation, the trained models were evaluated with respect to the estimated effective dose to the patient. The plot shows that a tight safety margin will result in a high radiation dose, as the imaging will be incomplete in many cases, requiring a repeat scan of the whole heart. A safety margin that is too large will not require a repeat scan, but will result in scans that cover a large area, thereby increasing the radiation dose. The graph shows that a safety margin of approximately 14 mm was optimal. *ED* expected effective radiation dose, *TOOD* task-aligned one-stage object detection, *VFNet* VariFocalNet.

All four networks performed well on the validation set, with only marginal differences (Table 3). Nearly all networks worked best when a safety margin of around 12–14 mm was added to both boundaries and achieved an expected ED of around 4.7 mSv (Fig. 4). Regarding accuracy, they all performed above 93% and obtained Dice scores around 90%. The mean absolute error (MAE) was slightly lower at the upper boundary than at the lower boundary.

**Table 3:** Results of the best-performing models in the validation set.

Model	Margin (mm)	Best learning rate	Expected effective dose ± SD (mSv)	Accuracy (%)	Dice coefficient ± SD (%)	MAE at upper boundary ± SD (mm)	MAE at lower boundary ± SD (mm)
Cascade-R-CNN	14	0.03	4.72 ± 4.08	97.6	90.3 ± 6.5	4.97 ± 4.05	5.74 ± 4.65
TOOD	12	0.009	4.70 ± 4.66	94.4	89.1 ± 7.5	5.25 ± 4.08	5.79 ± 4.93
VFNet	14	0.003	4.64 ± 3.88	98.0	89.9 ± 6.8	5.11 ± 4.14	5.96 ± 4.93
YOLO v11-N	14	Auto	4.97 ± 5.24	95.6	90.0 ± 7.2	5.27 ± 4.30	5.66 ± 5.01
YOLO v11-S	16	Auto	5.02 ± 4.78	97.2	90.3 ± 7.0	5.49 ± 4.34	5.84 ± 5.02
YOLO v11-M	14	Auto	5.04 ± 5.20	96.0	89.5 ± 7.4	5.65 ± 4.49	5.92 ± 5.03
YOLO v11-L	12	Auto	4.82 ± 4.82	93.2	89.3 ± 7.7	5.20 ± 4.25	5.66 ± 4.92
YOLO v11-X	14	Auto	4.88 ± 5.22	95.2	89.7 ± 7.4	5.28 ± 4.20	5.93 ± 5.05

The YOLO models were trained with an automatic learning rate schedule.

*MAE* mean absolute error, *SD* standard deviation.

Since the VFNet performed marginally better than the other networks, it was selected as the best-performing model.

### Testing

The VFNet (with the 14 mm safety margin and a learning rate of 0.003) was then retrained on all training data. The model was then evaluated on the independent test sets (Table 4). It showed an expected ED of 5.33 ± 6.42 mSv and 4.39 ± 4.23 mSv in the internal and external test sets, respectively. The Dice coefficients were 90.4% and 90.0%, leading to a high accuracy of 97.8% and 96.8% in the test sets. The clinical usefulness was slightly higher; considering the incomplete scans in detail, only 1 out of the 8 incomplete scans in the internal test set and 2 out of the 10 in the external test set would have obstructed diagnosis. A failure analysis showed no apparent reason why the predictions failed in these cases.

**Table 4:** Results in the two independent test sets.

Test set	Expected effective dose ± SD (mSv)	Estimated effective dose of the whole heart ± SD (mSv)	Reduction (%)	Accuracy (%)	Clinical usefulness (%)	Dice coefficient ± SD (%)	MAE at lower boundary ± SD (mm)	MAE at upper boundary ± SD (mm)
Internal	5.33 ± 6.42	11.35 ± 8.17	53.5	97.8	99.8	90.4 ± 6.2	5.16 ± 3.99	5.38 ± 4.11
External	4.39 ± 4.23	10.09 ± 8.0	56.4	96.8	99.3	90.0 ± 6.7	5.20 ± 3.96	5.78 ± 4.57

Results of the best-performing model (VFNet with 14 mm safety margin and a learning rate of 0.003) in the two independent test sets.

*MAE* mean absolute error, *SD* standard deviation.

### Dose reduction

Compared to the ED of the CT scan of the whole heart (11.35 ± 8.17 and 10.09 ± 8.0 mSv), the expected ED of the LAA scan was reduced by 53.5% and 56.4% (Table 4).

For the hypothesis that the dose reduction was larger than 33.3% compared to a full heart scan, we employed the Wilcoxon signed-rank test; the tests indicated that the reduction was statistically highly significant (*p* < 0.001).

## Discussion

A dedicated scan of the left atrial appendage (LAA) is often necessary to detect thrombi. Since it is challenging to delimit the LAA in the CT localizer, the whole heart is usually scanned in routine clinical practice, exposing the patient to unnecessary radiation. We presented an automation for delimiting the LAA in a CT localizer for a dedicated LAA scan using deep learning methods.

The model showed very high accuracy, approximately 98%, and could significantly reduce the effective dose to the patient. A dose reduction of about 55% (at least 5 mSv) was estimated compared to a whole heart CT scan. In addition, there were no large differences between the results of the validation and the two independent test sets, indicating that the model generalized sufficiently despite age and sex differences between the cohorts.

Instead of relying on delimitations directly in the localizer^10^, we employed a neural network to segment the corresponding CT and transferred the segmentation back to the localizer. The benefit of this approach is that the whole training process can be automated and does not require manual labeling of localizers. However, a slight disadvantage is that the annotations might not always be correct. Therefore, the model results were carefully checked by a board-certified radiologist. Since no systematic or unexpected errors were seen, this suggests successful training.

To determine whether our models could be applied in routine clinical practice, we used the expected effective dose for estimation, which considers repeated scans that occur in case of error, i.e., incomplete scans. Machine learning studies often consider metrics such as accuracy or overlap with the ground truth; however, such an approach would be suboptimal in our case because scanning the whole heart would always yield perfect accuracy but would ignore the radiation dose. Our expected ED reasonably considers both objectives and estimates the radiation dose to the patient when the model is used in clinical routine. Furthermore, since the network was trained on localizers from clinical routine rather than on mathematical projections of the CT scan, it can automatically compensate for the patient’s movement, different breathing, and heartbeat, to the extent that these were present in the training dataset. However, unforeseen variations may still occur, thus clinical supervision by a technologist is necessary. This challenge could be partially addressed by expanding the training dataset to include a broader and more diverse range of cases, thereby improving the model’s robustness. For seamless integration into routine practice, the network would need to be deployed directly on the CT scanner, which is currently only feasible for vendors. Given that the inference time for predictions is well below one second per image on mid-range hardware, the network would be suitable for clinical deployment, where a brief delay of several seconds is acceptable.

Automatic delimitations in the CT localizer to optimize the subsequent CT scan have recently been considered in the literature. Demircioğlu et al. used a conditional generative adversarial neural network to detect the lung area^10^ and showed that the network could produce scan ranges of high accuracy and reduce the radiation dose when compared to the technologist’s scan. It is likely that their approach could not achieve high accuracy when applied to the LAA because it relies on accurate delimitations in the localizer. Similarly, Zhang et al. delimited multiple regions using landmarks^11^. The advantage of their approach is that they can apply their method to several organs. Yet, because there are no landmarks in the localizer marking the LAA, their approach would not produce highly accurate delimitations. Salimi et al. proposed a network to delimit the chest^12^; however, they delimited the area on projections of the chest CT rather than on localizers, so their approach does not account for patient motion or respiratory effects and would not work optimally in clinical routine.

Other ways to reduce radiation exposure exist. In a systematic study, Hausleitner et al. identified multiple factors influencing the radiation dose in CCTAs, including patient weight, absence of stable sinus rhythm, tube current, and voltage. Also, they observed significant differences between study sites and CT systems^13^. However, only a few factors can be controlled; reducing the tube current and voltage might lower the image quality and lead to non-diagnostic imaging. Low-dose imaging could alternatively be post-processed: Kang et al. used a deep network to denoise low-dose scans, resulting in a quality similar to scans acquired at a higher dose^14^. Recently, photon-counting CT has been commercially introduced, which could potentially reduce the radiation dose^15,16^. Our approach is complementary to these methods since, in all of them, the LAA must be delimited, and would therefore lead to a larger decrease in radiation exposure.

We focused on the dose reduction of a single-pass LAA scan; however, it cannot reliably exclude the presence of thrombi. Transesophageal echocardiography (TEE) is often required as a second step^17^. Lazoura et al. showed that additional low-dose delayed CT scans of the LAA can be used instead of TEE^18^. Our approach is complementary to this technique and would further reduce the radiation dose.

Our study also aligns with previous works aiming to reduce radiation while improving accuracy. Ge et al.^19^ and Zhang et al.^20^ focus on reconstructing 3D structures from 2D images, reducing radiation exposure in clinical imaging. Similarly, Dai et al. use deep learning to track tumors with lower radiation doses in radiotherapy^21^. These studies, like ours, highlight the potential of deep learning to minimize radiation risk.

Our study has introduced a model for fully automated LAA scans and demonstrated that it performs well in two independent test cohorts. We did not apply any selection criteria to our test cohorts, which therefore include patients with and without pathology. For this reason, we expect the network to perform well in routine clinical practice.

Nevertheless, some limitations apply: our CT scans were all acquired on scanners from a single vendor and two nearby hospitals, which might limit the generalizability of the model. In addition, clinical routine sometimes requires a whole-heart scan, in which case the overall reduction in radiation exposure is relatively less. The same is true if the scan has to be repeated due to flow artifacts. A whole-heart scan might also include incidental findings^22^. Our annotations were produced by another neural network, which may have introduced minor segmentation errors. However, for both test sets, we reviewed the annotations and ensured that they contained no errors. We also estimated the ED by multiplying the scan length by the CTDIvol with a correction factor. A dedicated Monte Carlo estimation using phantoms may provide more accurate estimates.

Furthermore, this study employed the widely accepted Dice coefficient for evaluation. While it is known that it lacks sensitivity to smaller variations, which could be significant in this study due to the anatomical variability of the LAA, from a clinical perspective, the ED and clinical utility are the more critical metrics. These practical outcomes validate the network’s value in clinical practice, regardless of potential limitations in the Dice coefficient.

Our method attempts to account for patient movements between the acquisition of the localizer and the scan by incorporating a safety margin. However, in rare cases, larger movements may occur, especially if patients are larger than those in the study population, in which case the LAA scan could be incomplete. Therefore, supervision is required. Additionally, while the network has the potential to standardize the CT scan acquisition process compared to the inter-rater variability of technologists, the scan depends on many parameters that are not accounted for by the network.

Finally, we used off-the-shelf networks and only optimized the learning rate, which could be improved^23^. While the use of these networks may have limited the methodological innovation, they have proven efficient and reliable in our study, as demonstrated by their near-perfect clinical utility. Novel, more task-aligned network architectures could potentially improve performance slightly; however, given that the networks we used performed very similarly, it is reasonable to conclude that the current limitation lies in the study data. Thus, further development should be conducted on a larger, more heterogeneous population.

In conclusion, our study showed that a fully automated scan range delimitation for acquiring a CT of the left atrial appendage using a deep neural network is feasible. Compared to the acquisition of the whole heart, this approach can reduce radiation exposure to the patient by more than 50%.

## Methods

### Study data

This retrospective study was approved by the local ethics committee (Institutional Review Board of the University Hospital Essen; registry number 23-11244-BO). Due to the retrospective nature of the study, Institutional Review Board of the University Hospital Essen waived the need for obtaining informed consent. This study follows all relevant guidelines and regulations.

**Fig. 5:**
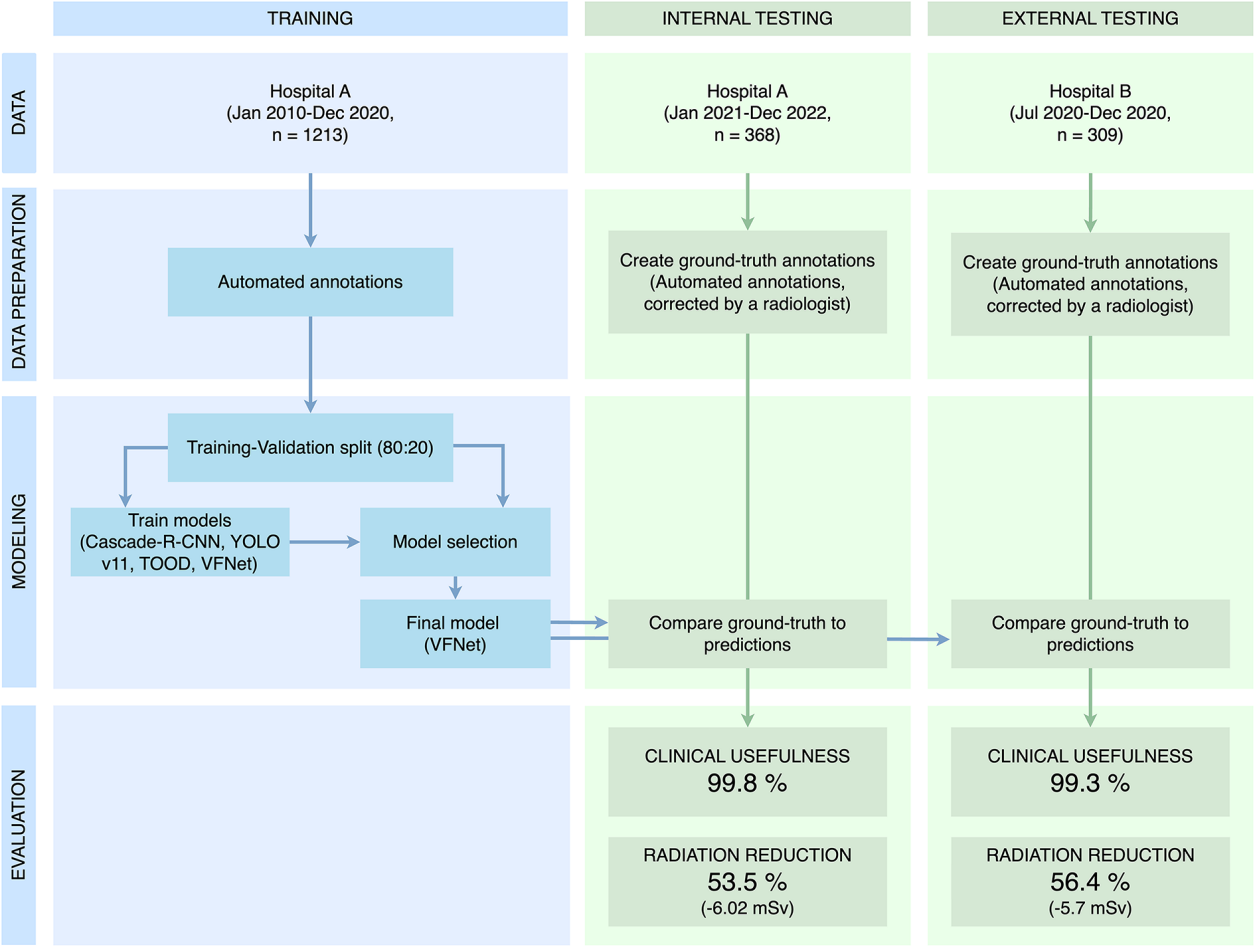
Study design. The aim of this study was to develop a network capable of delimiting the LAA in CT localizers, thereby automating the task usually performed by technologists. We used three independent cohorts from two hospitals. Due to the variation in the shape of the LAA, annotations from the CT scan were transferred back to the localizer. For model development, three networks were validated using a simple 80:20 split. The best-performing model in terms of the Dice coefficient was selected and tested in two independent cohorts. The model was then evaluated for clinical utility (or accuracy) and radiation dose compared to whole-heart scanning. *LAA* left atrial appendage, *TOOD* task-aligned one-stage object detection, *VFNet* VariFocalNet.

Three cohorts were collected retrospectively in anonymized form by querying the radiological information system and the picture archiving and communication system: a training cohort, an internal validation cohort, and an external test cohort. The overall study design is shown in Fig. 5.

For the training cohort, 1500 CT examinations of patients who underwent a coronary contrast-enhanced CT on second-generation dual-source CT scanners were randomly selected (University Hospital Essen, Germany; between January 2010 and December 2020). CT examinations were only included if a corresponding CT localizer was found. Examinations of minors (< 18 years) were excluded. In addition, to ensure homogeneity of the data, examinations were excluded if the corresponding localizer had a pixel spacing different from 1.0 mm, which is the default pixel spacing in the clinical routine. Also, if the heart was not fully acquired in the CT, the examination was excluded. Similarly, an internal test cohort was collected: 500 randomly selected CT scans between January 2021 and December 2022 were included with similar criteria as the training cohort. During collection, it was ensured that no patient from the training set was included in the test set to avoid bias. Finally, an external test cohort from a collaborating hospital (Elisabeth Hospital, Essen, Germany) was collected with the same criteria between July 2020 and December 2020. A board-certified radiologist visually reviewed CT scans and localizers to ensure the image quality met the required standards.

### CT scan acquisition

CT localizers and scans were acquired during inspiration in the anterior-posterior direction on modern multi-slice CT scanners (Siemens Healthineers) (Table 2). Acquisition of the CT was performed using automated tube current adaptation, using a prospective adaptive triggering primarily (quality reference mAs: 370 mAs (Flash)/300 mAs (Force)). An automated tube current modulation (Siemens CARE Dose 4D, Siemens Healthineers) was used for all examinations.

### Scan range delimitation

**Fig. 6:**
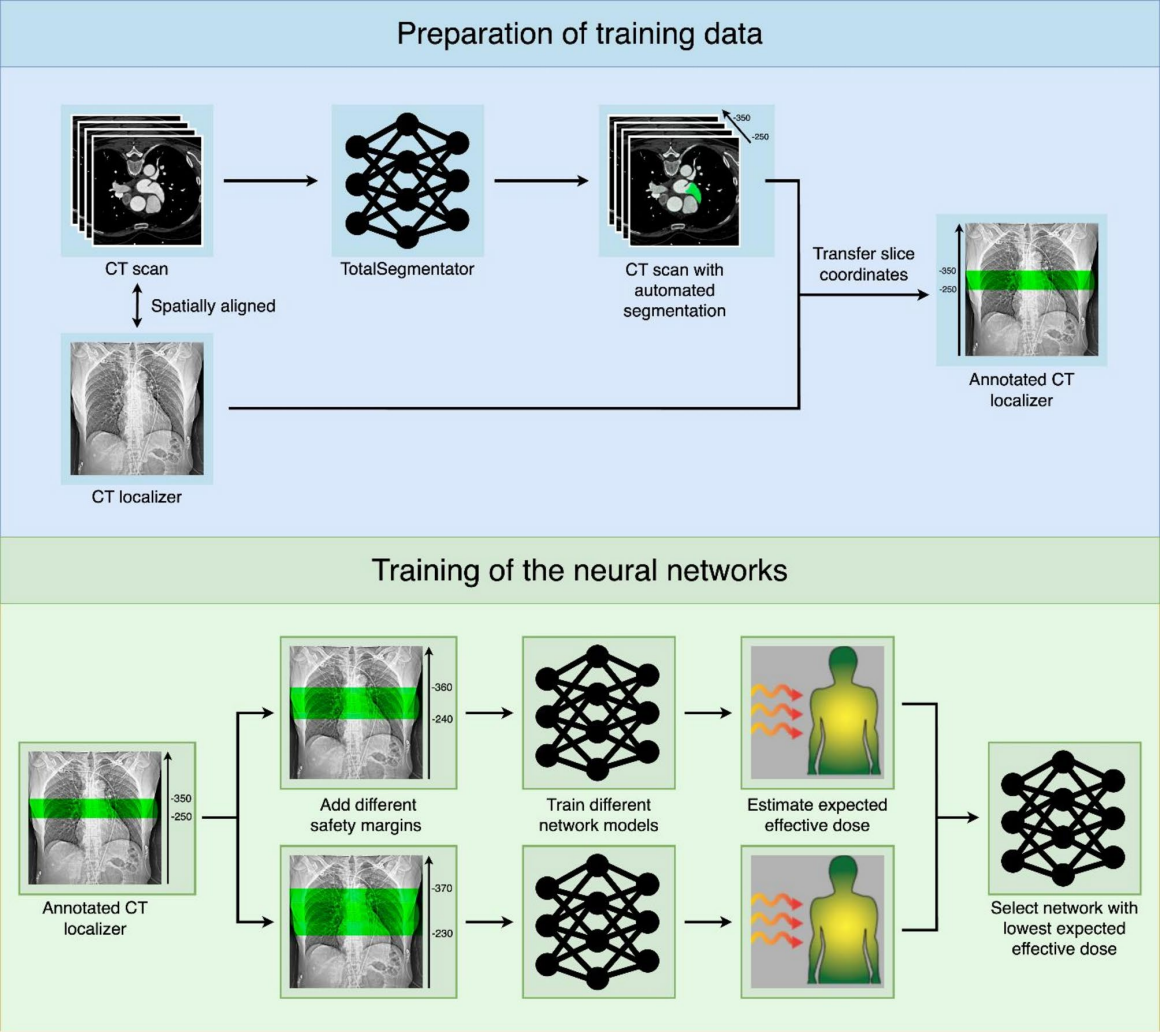
Workflow for network training and model selection. CT scans were automatically segmented using the publicly available software TotalSegmentator to identify the left atrial appendage (LAA). Slice coordinates defining the LAA region were then transferred to the corresponding CT localizers used to acquire the scans. To account for patient motion during acquisition, multiple safety margins were incorporated during the training of the network models. The model achieving the lowest expected effective dose (on the validation set) was selected as the optimal network.

Since the LAA is not easily delimited in the CT localizer due to its different anatomical shapes^24^, the CT scans were used instead. We used publicly available segmentation software based on deep learning^25^ to segment the LAA in all CT scans. The slice coordinates of the LAA region were then determined and transferred back to the localizer to obtain the delimitation (Fig. 6). Note that the annotations in the localizer may not be visually consistent due to missing landmarks and patient motion, but they would result in a perfect annotation in the CT scan.

For the test cohorts, all segmentations of the LAA on the CT scans were reviewed and corrected when necessary to avoid errors in the ground truth.

### Safety margins

Accurate annotation of the LAA in the localizer would be strongly affected by any movement of the patient (for example due to breathing) and may lead to incomplete imaging. In clinical practice, a safety margin is added to the annotation, e.g., instead of delimiting the region from the carina to the apex at both boundaries, a margin of approximately 1 cm is added. However, the size of the margin is not well-defined and depends strongly on the personal preference of the technologist. Thus, instead of fixing such a margin a priori, we considered different margins during training and selected the one that yielded the best performance (Fig. 6).

### Neural network training

The training data were further split randomly into a training and validation set (80:20). Then, four network architectures were trained to detect the scan range delimitation: The Cascade-R-CNN^26^, the VariFocalNet (VFNet)^27^, the Task-aligned One-stage Object Detection (TOOD) network^28^, and the You Only Look Once v11 (YOLO) network^29^. All networks were pretrained using the ‘Common Objects in Context’ dataset^30^. The networks were then evaluated based on the expected mean effective radiation dose in the validation split, which estimates the patient’s radiation exposure during routine clinical practice if the model’s predictions were to be used. In addition, the accuracy (measuring whether the LAA would be fully acquired in the corresponding CT), the Dice coefficient, and the absolute difference (in mm) of the total scan length, the absolute difference at the upper and lower boundaries, and the number of potentially incomplete scans were computed. The model with the best-performing parameters was then retrained on the whole training set and evaluated in the test cohorts.

Implementation details can be found in the Supplementary Materials.

### Testing

Both test cohorts were evaluated similarly to the validation set. In addition, all error cases (defined as a prediction that resulted in an incomplete CT) were reviewed by a board-certified radiologist (K.N. with 20 years of experience and expertise in cardiac CT) to determine whether they would have obstructed the diagnosis, which we defined as clinical usefulness.

### Expected effective radiation dose

We estimated how much the automatically determined scan range could reduce the radiation exposure to the patient. In clinical routine, the technologist would use the network prediction first and check for completeness. If the imaging of the LAA was complete, the patient was exposed to the effective radiation dose (ED) for the corresponding LAA scan. However, if the imaging was not complete, the technologist would redo the acquisition, but since the network failed, they would scan the whole heart. Therefore, the ED to the patient would be the sum of the LAA and the whole-heart scan. In addition, in the rare event that the network fails to produce a result (which is technically possible), the technologist would proceed with scanning the whole heart. This combined ED is referred to as the expected effective radiation dose.

The ED of each scan was estimated by multiplying the scan length by the volumetric computed tomography dose index (CTDIvol), corrected by a factor k. This factor was set to 0.026 mSv·mGy^− 1^cm^− 1^ for males and 0.045 mSv·mGy^− 1^cm^− 1^ for females to account for breasts^31^. These values correspond to the lower limit of the 95% CI determined in a recent study^32^.

### Statistical methods

Differences in demographics were assessed by using Chi-square or t-tests. Descriptive values were reported as mean ± standard deviation. The evaluation of radiation exposure is based on the assumption that using the shorter scan range for the LAA will reduce radiation exposure by at least 1/3 compared to the radiation exposure of the whole heart scan. Thus, a one-sided Wilcoxon signed-rank test for superiority was conducted. *P* values below 0.05 were considered significant. Statistical analyses were conducted using the statsmodel library in Python 3.8.

## Electronic supplementary material

Below is the link to the electronic supplementary material.

Supplementary Material 1

## Data Availability

The code is available in the GitHub repository at https://github.com/aydindemircioglu/LAA. The datasets used and/or analyzed during the current study are available from the corresponding author upon reasonable request.
