# Supplementary material for: Detecting the left atrial appendage in CT localizers using deep learning
Source: Sci Rep. 2025 May 2;15:15333. doi: 10.1038/s41598-025-99701-6 (PMC12048584; doi:10.1038/s41598-025-99701-6)
Supplement: Supplementary file 1 — Supplementary Material 1 [file 41598_2025_99701_MOESM1_ESM.docx]

**Detecting the Left Atrial Appendage in**

**CT Localizers Using Deep Learning**

**Supplemental Materials**

**Preprocessing**

All localizer images had a uniform size of 512x512 pixels. They were converted from a 16-bit greyscale to an 8-bit pseudo-RGB by adding two different contrast-limited adaptive histogram equalization^1^ to the image as red and green channels. This was necessary because the networks were pretrained on RGB images. Images were then normalized to the intensity mean and standard deviation of the ‘Common Objects in Context’ dataset.^2^

**Neural Network Training**

Four supervised network architectures were employed for the detection of the scan range: The Cascade-R-CNN^3^, the VariFocalNet (VFNet)^4^, the Task-aligned One-stage Object Detection (TOOD) network^5^, and the You Only Look Once v11 (YOLO) network^6^. All implementations were taken from the MMDetection library^7^ except for YOLO, where the python module ultralytics was used. These networks were chosen because they were either the best-performing models in the MMDetection model zoo (see <https://mmdetection.readthedocs.io/en/latest/model_zoo.html>), or, in case of YOLO, they are known to perform very well. The weights were initialized using the weights obtained by pretraining on the ‘Common Objects in Context’ dataset.^2^ Different safety margins (4, 6, 8, …, 20 mm) were considered during training. Details on the networks can be found in the MMDetection framework that was employed for development. Details on the experiment can be found in the study repository. (Code is available on the GitHub repository at <https://github.com/aydindemircioglu/LAA>). The network was developed using Python 3.8, MMDetection 3.00 and ultralytics 8.3.82.^7^

**Cascade R-CNN**

Images were augmented using random cropping to a size of 480x480, followed by rescaling to 1333x800. Random brightness adjustments (±1/16), contrast and saturation variations (±20%), Gaussian noise, elastic transformations, and random flips were applied. The batch size was set to 4.

The learning rate schedule consisted of a linear warm-up phase with a start factor of 0.001 for the first 500 iterations, followed by a multi-step decay with milestones at epochs 8 and 11, reducing the learning rate by a factor of 0.1. Training was conducted for a total of 12 epochs. Optimization was performed using SGD with a momentum of 0.9, and a weight decay of 0.0001. The learning rate was selected from {0.09, 0.03, 0.009, 0.003, 0.0009, 0.0003}.

The training was conducted using a Cascade R-CNN model with a ResNet-50 backbone and an FPN neck. The loss function combined cross-entropy for classification and Smooth L1 loss for bounding box regression.

**VariFocalNet**

Images were augmented using random cropping to a size of 480x480, with a subsequent rescaling, random brightness (±1/16), and random contrast and saturation changes (± 20%). In addition, Gaussian noise was added, and a random elastic transformation was applied. The batch size was set to 4.

The learning rate schedule consisted of a linear warm-up phase with a start factor of 0.1 for the first 500 iterations, followed by a multi-step decay with milestones at epochs 16 and 22, reducing the learning rate by a factor of 0.1. Training was conducted for a total of 24 epochs. Optimization was performed using SGD with a momentum of 0.9, and a weight decay of 0.0001. The learning rate was chosen from {0.09, 0.03, 0.009, 0.003, 0.0009, 0.0003}.

The training was conducted using a VFNet model with a ResNeXt-101 backbone and an FPN neck. The loss function combined Varifocal Loss for classification and GIoU loss for bounding box regression, with an additional refined GIoU loss

**Task-aligned One-stage Object Detection**

Images were augmented using random cropping to a size of 480x480, followed by rescaling to 1333x800. Random brightness adjustments (±1/16), contrast and saturation variations (±20%), Gaussian noise, elastic transformations, and random flips were applied. The batch size was set to 4.

The learning rate schedule consisted of a linear warm-up phase with a start factor of 0.001 for the first 500 iterations, followed by a multi-step decay with milestones at epochs 16 and 22, reducing the learning rate by a factor of 0.1. Training was conducted for a total of 24 epochs. Optimization was performed using SGD with a momentum of 0.9 and a weight decay of 0.0001. The learning rate was chosen from {0.09, 0.03, 0.009, 0.003, 0.0009, 0.0003}.

The training was conducted using a TOOD model with a ResNeXt-101 backbone and an anchor-free head. The loss function combined Quality Focal Loss for classification and GIoU loss for bounding box regression.

**YOLO v11**

YOLO v11 uses several different augmentations, including random cropping, scaling, and flipping, along with color jittering for brightness, contrast, and saturation variations. Additional augmentations included noise, erasing and mosaic augmentation to enhance generalization. The default augmentations were not changed, and the details can be found in the source code of ultralytics. For training, the batch size was set to 16 for the N, S, M models and 8 for the L and X models.

The optimizer was set to ‘auto’, using AdamW with a momentum of 0.9. Training was conducted for a total of 100 epochs.

For training, YOLO used a combined binary cross-entropy loss function.

**Model selection**

The training data were further split randomly into a training and validation set (80:20). The training split was used for training the network while the validation split was used to determine the best-performing model.

The best-performing model was selected based on the expected mean effective radiation dose in the validation cohort, which estimates the patient's radiation exposure during routine clinical practice if the model's predictions were to be used. In addition, to judge the quality of the training, the accuracy (measuring whether the LAA was acquired fully in the corresponding CT), the Dice coefficient between the predicted and the ground truth annotation, the absolute difference (in mm) of the total scan length, the absolute difference at the upper and lower boundaries, and the number of potential incomplete scans was computed.

The model with the best-performing parameters was then retrained on the entire training set (without a validation split). This final model was then evaluated on the internal and external test cohorts, neither of which was used in any way during training and model selection.

**Experimental hardware**

All experiments were conducted on a workstation running Ubuntu 22.04.5 LTS and equipped with an AMD Threadripper 2950X with 128 GB of RAM and a Nvidia TITAN RTX graphics card with 24 GB of VRAM.

**References**

1. Pizer SM, Amburn EP, Austin JD, et al. Adaptive histogram equalization and its variations. *Computer Vision, Graphics, and Image Processing*. 1987;39(3):355-368. doi:10.1016/S0734-189X(87)80186-X

2. Lin TY, Maire M, Belongie S, et al. Microsoft COCO: Common Objects in Context. *arXiv:14050312 [cs]*. Published online February 20, 2015. Accessed March 21, 2022. http://arxiv.org/abs/1405.0312

3. Cai Z, Vasconcelos N. Cascade R-CNN: High Quality Object Detection and Instance Segmentation. *IEEE Transactions on Pattern Analysis and Machine Intelligence*. 2021;43(5):1483-1498. doi:10.1109/TPAMI.2019.2956516

4. Zhang H, Wang Y, Dayoub F, Sunderhauf N. VarifocalNet: An IoU-Aware Dense Object Detector. In: ; 2021:8514-8523. Accessed March 21, 2022. https://openaccess.thecvf.com/content/CVPR2021/html/Zhang_VarifocalNet_An_IoU-Aware_Dense_Object_Detector_CVPR_2021_paper.html

5. TOOD: Task-aligned One-stage Object Detection. Accessed May 30, 2023. https://www.computer.org/csdl/proceedings-article/iccv/2021/281200d490/1BmEvqSJIEE

6. Glenn Jocher, Jing Qiu, Ayush Chaurasia. Ultralytics YOLO. Ultralytics. January 10, 2023. https://ultralytics.com

7. Chen K, Wang J, Pang J, et al. MMDetection: Open MMLab Detection Toolbox and Benchmark. *arXiv:190607155 [cs, eess]*. Published online June 17, 2019. Accessed November 10, 2020. http://arxiv.org/abs/1906.07155
